# A novel alkane monooxygenase (*alkB*) clade revealed by massive genomic survey and its dissemination association with IS elements

**DOI:** 10.7717/peerj.14147

**Published:** 2022-09-28

**Authors:** Shaojing Wang, Guoqiang Li, Zitong Liao, Tongtong Liu, Ting Ma

**Affiliations:** College of Life Sciences, Nankai University, Tianjin, China

**Keywords:** Alkane monooxygenase, *alkB*, *alk*-gene clusters, Phylogenetic diversity, IS elements, Niches distribution

## Abstract

**Background:**

Alkanes are important components of fossil energy, such as crude oil. The alkane monooxygenase encoded by *alkB* gene performs the initial step of alkane degradation under aerobic conditions. The *alkB* gene is well studied due to its ubiquity as well as the availability of experimentally functional evidence. The *alkBFGHJKL* and *alkST* clusters are special kind of *alkB*-type alkane hydroxylase system, which encode all proteins necessary for converting alkanes into corresponding fatty acids.

**Methods:**

To explore whether the *alkBFGHJKL* and *alkST* clusters were widely distributed, we performed a large-scale analysis of isolate and metagenome assembled genome data (>390,000 genomes) to identify these clusters, together with distributions of corresponding taxonomy and niches. The set of *alk*-genes (including but not limited to *alkBGHJ*) located near each other on a DNA sequence was defined as an *alk*-gene cluster in this study. The *alkB* genes with *alkGHJ* located nearby on a DNA sequence were picked up for the investigation of putative *alk*-clusters.

**Results:**

A total of 120 *alk*-gene clusters were found in 117 genomes. All the 117 genomes are from strains located only in *α*- and *γ*-proteobacteria. The *alkB* genes located in *alk*-gene sets were clustered into a deeply branched mono-clade. Further analysis showed similarity organization types of *alk*-genes were observed within closely related species. Although a large number of IS elements were observed nearby, they did not lead to the wide spread of the *alk*-gene cluster. The uneven distribution of these elements indicated that there might be other factors affecting the transmission of *alk*-gene clusters.

**Conclusions:**

We conducted systematic bioinformatics research on *alk*-genes located near each other on a DNA sequence. This benchmark dataset of *alk*-genes can provide base line for exploring its evolutional and ecological importance in future studies.

## Introduction

Alkanes are widely distributed in nature, not only as the most abundant components of crude oil, but also low concentration alkanes produced by plants, insects, and microorganisms. It has been reported that marine algae could produce about 308 to 771 million tons of alkanes annually (Lea-Smith et al., 2015; Schirmer et al., 2010; Valentine & Reddy, 2015). Alkanes can serve as chemo-attractants as well as substrate. The capacity to use hydrocarbons as sole carbon and energy source is very common, and not restricted to any particular group of microorganisms, owing to the wide spread of alkane hydroxylase system (Nie et al., 2014). According to alkanes which could be catalyzed by responsible enzymes, they have been broadly classified into short-, mid-, and long-chain alkane degradation systems. The integral membrane non-heme iron monooxygenase, which catalyzes the initial hydroxylation of mid-chain alkanes, is a kind of widespread alkane degradation enzyme encoded by *alkB* (Rojo, 2009; Van Beilen et al., 2002). It has been well studied due to its ubiquity as well as the availability of experimentally functional evidence (Wang & Shao, 2013). To date, quite a few studies describing *alkB* sequences and their phylogenetic structure were elucidated (Nie et al., 2014; Smith et al., 2013; Wang et al., 2010; Wang & Shao, 2013). The *alkB* gene was generally considered as a molecular marker for alkane degradation as well (Christian et al., 2020).

The *alkB*-type alkane hydroxylase system consists of three core components: an integral membrane alkane monooxygenase and two soluble proteins (rubredoxin and rubredoxin reductase) which act as electron carriers between NADH and the hydroxylase (Nieder & Shapiro, 1975). The *alkBFGHJKL* and *alkST* clusters were a special kind of *alkB*-type alkane hydroxylase system. They were initially reported on OCT-plasmid of *Pseudomonas putida* PGo1 (Eggink et al., 1987a; Eggink et al., 1987b; Van Beilen et al., 1992; Van Beilen, Wubbolts & Witholt, 1994). The *alk*-gene clusters encode all proteins necessary for converting alkanes into corresponding fatty acids, endowing microorganisms with the ability to utilize alkanes as the sole carbon and energy source. At present, bioinformatics analysis of *alkB* genes on gene-cluster level has not been reported.

The *alkB*-type alkane hydroxylase system from the plasmid of *Pseudomonas putida* GPo1 was unusual not only because of its compact gene organization, but also as its n-alkane substrate range, from C_3_ to C_12_ (alkanes in gasoline range), while many other related hydroxylases typically oxidize alkanes longer than C_10_ (Tsai et al., 2017). Schneiker et al. (2006) reported *alkSBGHJ* operon and *alkK* nearby in *Alcanivorax borkumensis* SK2, a plasmid free strain containing only a single circular chromosome, suggesting *alk*-gene clusters were not restricted in plasmid and neither in *P. putida*.

So far, many comprehensive studies have been carried out to investigate *alk*-genes, including functional characterization (Smits, Witholt & Van Beilen, 2003), regulation mechanism (Canosa et al., 2000), fusion and transfer (Chakrabarty, 1973), heterologous expression (Liu et al., 2011; Liu et al., 2010; Minak-Bernero et al., 2004; Sameshima et al., 2008; Smits et al., 2001; Wang et al., 2017; Whyte et al., 2002), promoter (Staijen Ivo, Marcionelli & Witholt, 1999) and biotransformation application (Lu et al., 2021; Van Nuland et al., 2017). Sequence analysis of *alk*-gene clusters had observed several insertion sequences (IS) located in its flanking regions. Together with its lower G-C content compared with the host strain, previous studies suggested these genes were part of a large mobile element, which may be responsible for horizontal transfer across different species (Van Beilen et al., 2001). Although it was supposed to spread horizontally, there was still a lack of relevant research on whether it is widely distributed.

To fill this knowledge gap, we performed a large-scale analysis of isolate and metagenome assembled genome data to identify *alk*-gene clusters, together with distributions of corresponding taxonomy and niches. A comprehensive bioinformatics analyses, including phylogenetic analysis, taxonomy distribution, niches distribution, organization structure, chromosome location and IS elements analyses, were performed to provide detailed information of *alkB* genes on gene-cluster level.

## Materials and Methods

### Identification of *alk*-gene cluster

An initial database of *AlkBFGHJKL*, *alkN* and *alkST* (alk-genes hereafter) was constructed using protein sequences described in the study by Van Beilen et al. (2001). All available prokaryotes genomes (both bacteria and archaea) were downloaded from NCBI (http://www.ncbi.nlm.nih.gov) on March 2, 2022, representing for 200 phyla (146 out of which were candidatus). More specifically, the genome sequences of over 390,000 isolates or MAGs from 57,335 species belonging to 3,687 genera were included in this analysis. At the same time, the taxonomy and niches information corresponding to these genomes were also downloaded. The sequences from genomes with assembly levels of “Complete” or “Chromosome” were marked as chromosomal or plasmid according to their corresponding description. The rest genome sequences were marked as “Contig/Scaffold”. Subsequently, gene prediction was performed using Prokka v1.13 (Seemann, 2014). The presence of each gene in the genome was screened using blastp with an e-value of 1e−10 (Camacho et al., 2009).

Identification of putative gene clusters was performed using a two-step method. For the first, a set of four genes, that were *alkBGHJ* were used as markers for identification of putative *alk*-fragment (DNA fragment containing *alk*-genes) location using criteria as follows: (1) *alkB* must exist, (2) *alkGHJ* were present within 10 kb flanking region of *alkB* gene, (3) sequence of DNA segments flanking the *alkB* gene, ranging from 10 kb upstream to 28 kb downstream of the starting codon, were extracted from genome sequence for next step. Second, *alk*-fragments were re-predicted using prokka (Seemann, 2014), and then screened by each gene of *AlkBFGHJKL*, *alkN* and *alkST* using blastp in blast package v2.9.0 (Camacho et al., 2009). All *alk*-fragments were verified manually and the fake ones were deleted. The *alk*-fragments at the end of contigs, which contain truncated gene clusters, were excluded in this analysis. The IS elements in *alk*-fragments were identified using ISEScan v1.7.2.2 (Xie & Tang, 2017). Organization of *alk*-genes and IS elements were plotted using R v4.1.2 (R Core Team, 2021) with ggplot.

### Phylogeny of *alk*-gene cluster-carrying genomes

To build the reference phylogenetic tree for this analysis, bcgTree v1.2.0 (Ankenbrand & Keller, 2016) was performed. The bcgTree pipeline builds phylogenetic trees from bacterial core genomes, these were 107 essential genes. Briefly, gene prediction was performed by prodigal v2.6.3 (Hyatt et al., 2010), then the core genes were identified using hmmsearch in HMMER v3.2.1 (Eddy, 1998; Eddy, 2009) and aligned using muscle v3.8.31 (Edgar, 2004). The unreliable alignment regions were removed by using Gblocks v0.91b (Castresana, 2000). The resulting alignment was used to construct a maximum likelihood phylogenetic tree using the RAxML v8.2.12 (Stamatakis, 2014) with rapid bootstrap 1000. The final tree was midpoint rooted and visualized using iTOL (Letunic & Bork, 2021).

### Phylogeny of the *alkB* genes

To infer the phylogenetic structure of the *alkB* gene, we manually downloaded nucleotide sequences coding alkane 1-monooxygenase from KEGG (http://www.kegg.jp) with ortholog number K00496, along with corresponding taxonomy information for each sequence. The *alkB* genes with identical nucleotide sequences and taxonomy compared with *alkB* genes in *alk*-gene clusters were eliminated.

The retained *alkB* gene sequences from KEGG (http://www.kegg.jp) and the *alkB* gene sequences retrieved from *alk*-fragment were combined for phylogenetic analysis. All the *alkB* gene sequences were aligned using mafft v7.407 (Katoh & Standley, 2013). And then maximum likelihood phylogenetic tree was constructed using the RAxML v8.2.12 (Stamatakis, 2014) with rapid bootstrap 1000. The *xylM*, which encodes the xylene monooxygenase subunit, was used as the outgroup, as described in the studies by Nie et al. (2014) and Karthikeyan et al. (2021).

### Organization structure of *alk*-genes

The sequence fragments were extracted from the genome sequence for each *alk*-gene cluster, from 10kbp upstream of the *alkB* gene to 28kbp downstream, using inhouse perl scripts. These sequence fragments were defined as *alk*-fragments. The corresponding gene prediction and annotation information were extracted at the same time. The organization structure was plotted using ggplot2 and gggenes packages in R v4.1.2.

## Results

### Phylogenetic diversity of genomes carrying *alk*-gene clusters

**Figure 1:**
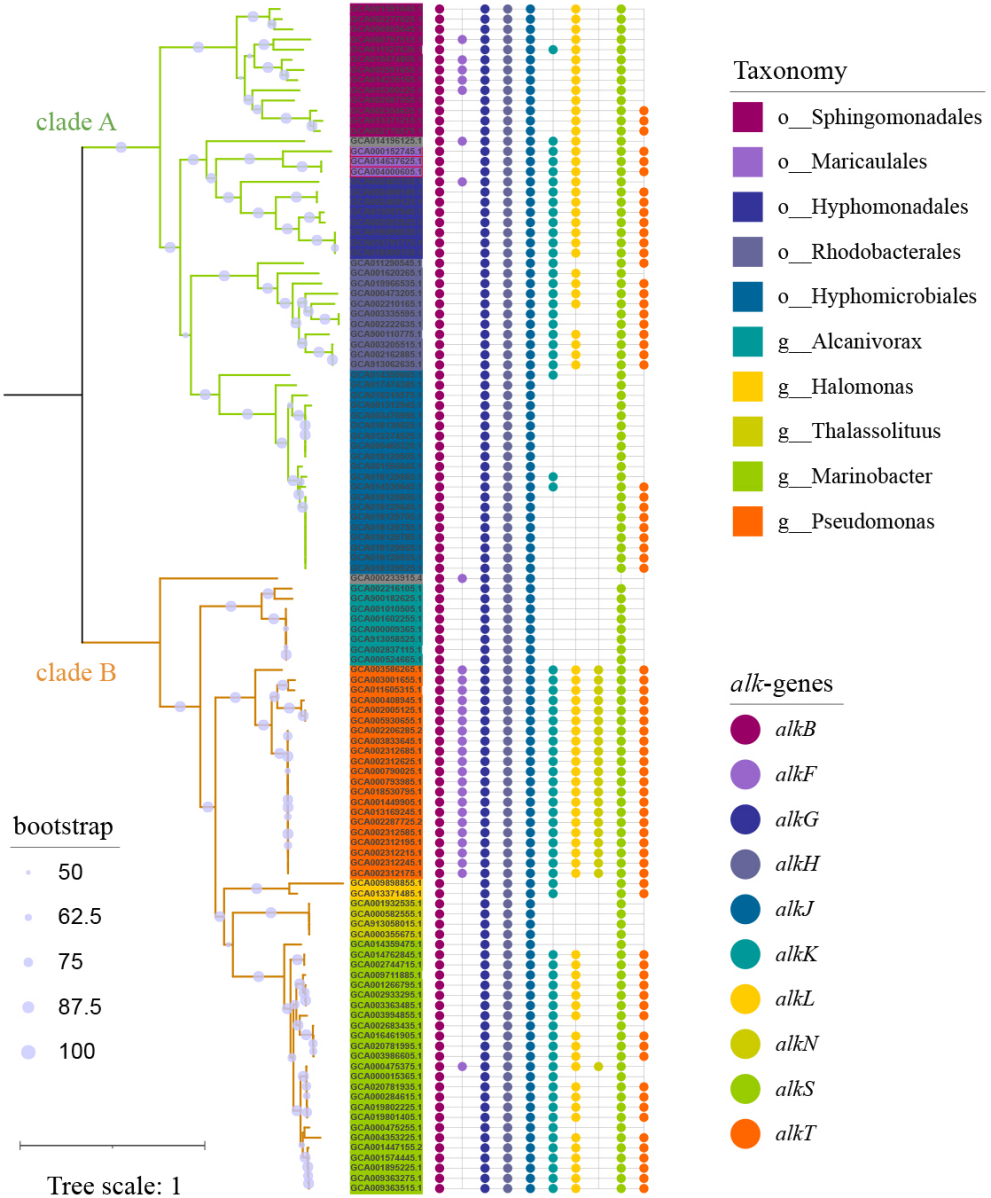
Phylogenetic tree of strains carrying *alk*-gene clusters. The Maximum Likelihood phylogenetic tree was constructed from core genes with seqboot 1000. The green branch represented for clade A, which was composed of strains from *α*-proteobacteria. The yellow branch represented for clade B, which was composed of strains from *γ*-proteobacteria. The corresponding taxonomy was represented by the background colors of leaf labels. The Bubble diagram on the right represents the presence of *alk*-genes.

To capture as much diversity as possible, >390 000 genomes which include both MAGs and isolate genomes, were screened for the presence of *alk*-gene clusters. Totally 117 genomes (Table S1) carrying at least one set of *alk*-gene clusters were observed, which formed 2 distinct clades (Fig. 1). Clade A was composed of 56 strains, all in *α*-proteobacteria at class level, representing for 6 orders and at least 26 genera. While clade B was composed of 61 strains, all in *γ*-proteobacteria at class level, representing for at least 3 orders and 6 genera. Strains in genus *Pseudomonas,* located in clade B, had consistent and complete *alk*-genes sets. While all the other species had incomplete *alk*-genes set, more or less. Longer branch length reflected distant phylogenetic relationship between species in clade A, suggesting wider distribution of *alk*-gene clusters compared with those in clade B.

### Niches distribution of strains carrying *alk*-gene clusters

These *alk*-gene clusters carrying strains had extensive niches distribution, including marine, soil, water, oil field and human as inferred in Fig. S1. But the niches distribution has a strong correlation with taxonomy, rather than *alk*-genes. Soil niched strains were enriched in *α*-proteobacteria, accounting for the major of *Sphingomonadales* and *Hyphomicrobiales* in clade A. Freshwater niched strains were limited in *α*-proteobacteria. While urban water niched strains enriched in *γ*-proteobacteria, account for almost half of *Pseudomonas*. It is speculated that *alk*-gene clusters had less impact on niches adaptation. On the other hand, they had the potential for dissemination in a wide range of environments.

### *Alk*-fragments distribution on genome

Totally 120 *alk*-fragments were extracted from 117 genomes, as two-copies of *alk*-fragments exist in three genomes. All of the three two-copies *alk*-fragments carrying strains are located in clade B (*γ*-proteobacteria). *Marinobacter nauticus* VT8, a species of *Marinobacter* isolated from oil producing well, had both *alk*-fragments on a single chromosome, separated by ∼150 kb distance. The other two strains were *Marinobacter salarius* strain HL2708#2, a species of *Marinobacter* isolated from Lava and *Alcanivorax* sp. N3-2A, a species of *Alcanivorax* isolated from coastal sediments, had one *alk*-fragment on chromosome and the other on plasmid.

Among all of the 120 *alk*-fragments observed, there were 24 cases located on chromosome, while only 5 cases were on plasmid. And the remaining 91 cases were located on contig or scaffold, owning to assemble level. Although *alk*-gene clusters were initially found on plasmids, they were also observed on chromosomes (Fig. S2).

### Phylogenetic diversity of *alkB* from *alk*-fragments

**Figure 2:**
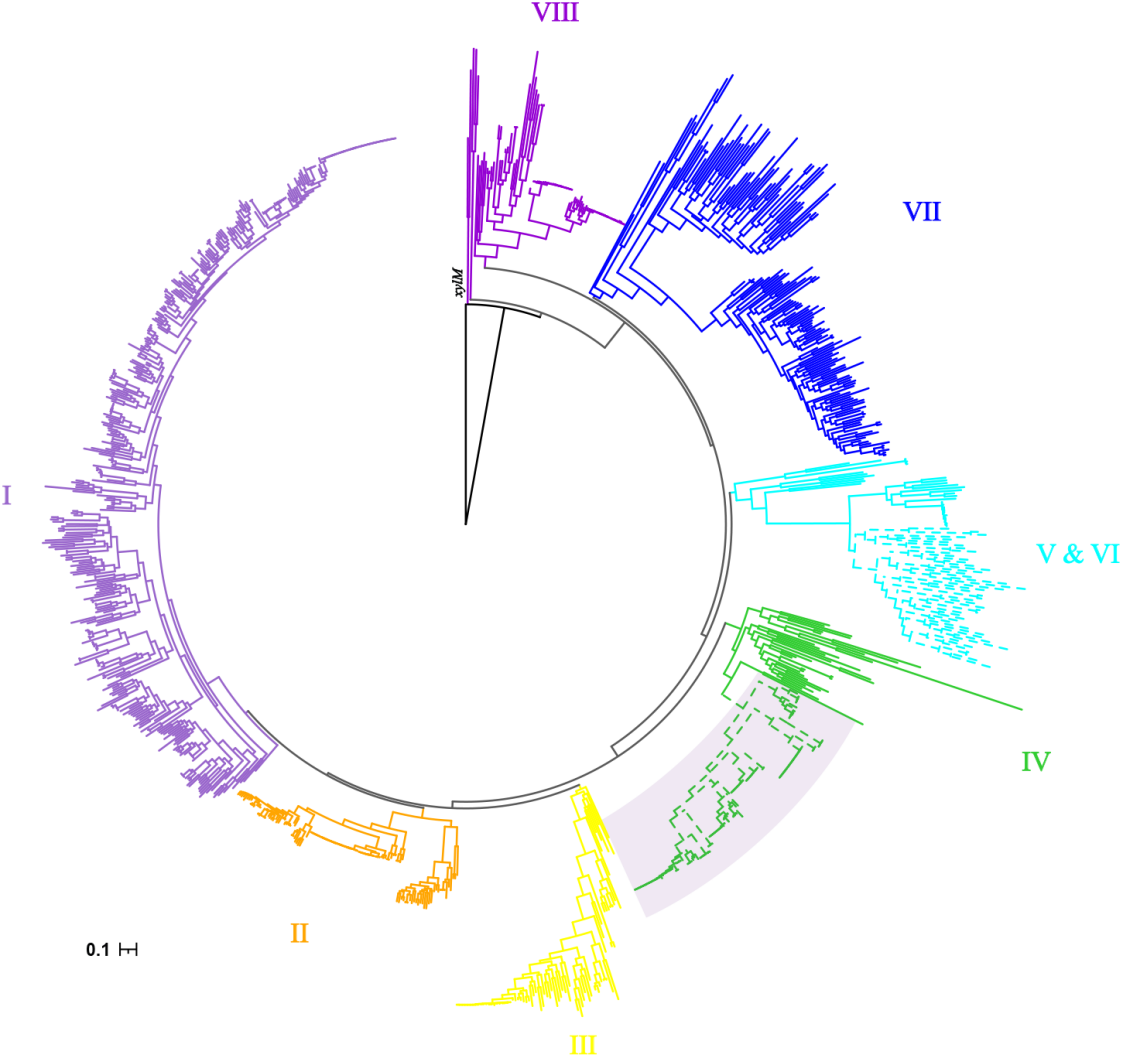
Phylogenetic tree of *alkB* genes. The Maximum Likelihood phylogenetic tree was constructed from the nucleotide sequence of *alkB* genes with seqboot 1000. The *xylM* gene was used as outgroup. The cluster IDs were consistent with those reported by Nie, with branches colored by solid color. The *alkB* genes extracted from *alk*-fragments were marked with a fan-shaped shadow background.

As previously reported by Nie, *alkB* genes were clustered into eight clusters (Nie et al., 2014). The cluster IV had more taxonomy diversity, which were from *α*-, *β*- and *γ*-proteobacteria. Consistent phylogenetic structure of *alkB* genes was obtained in this study, as shown in Fig. 2. Interestingly, the 120 *alkB* genes located in *alk*-gene clusters were limited to a mono-clade, which formed a subclade of cluster IV.

As mentioned above, *alk*-gene clusters contained a complete gene set for alkane degradation, representing for lower adaptability requirements of the recipient in the transfer process between species, suggesting its potential for wider dissemination. But the results here showed that the spread of *alk*-gene clusters was limited. Their phylogenetic relationship was very conservative. Together with its limitation within a certain class of species as results described above, it was suggested that there might be restrictive factors which limit its dissemination.

### Organization diversity of *alk*-genes and IS elements distribution

**Figure 3:**
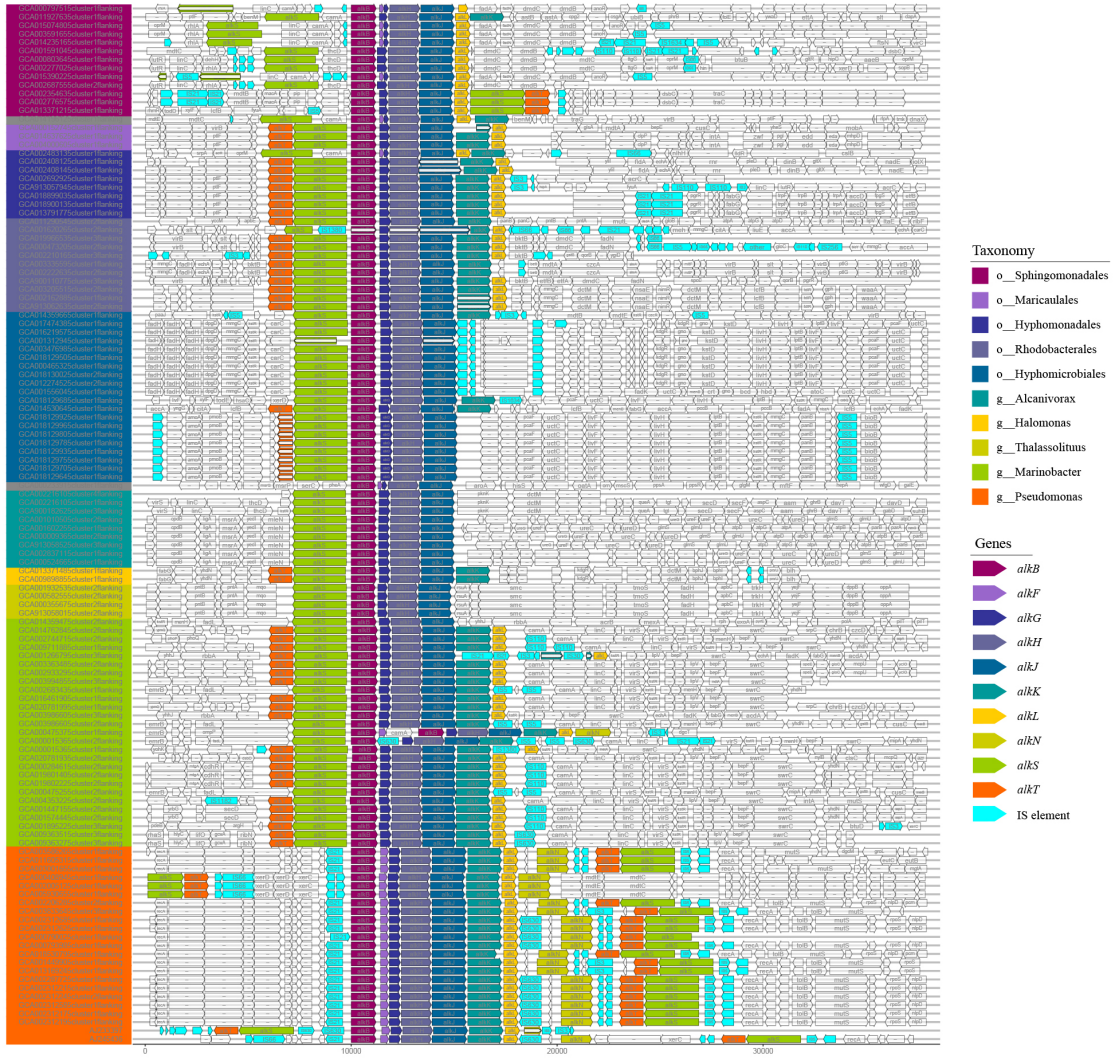
Comparative analysis of *alk*-genes organization structure. These fragments were ordered according to the corresponding strains shown in the phylogenetic tree of Fig. 1. These gene clusters were aligned according to the starting code of *alkB*. The nested white bars inside represented for pseudogenes.

The organization diversity of *alk*-genes was related to taxonomy lineage, as inferred from Fig. 3. Species with similar genetic relationships showed similar organization types of *alk*-genes. The *alk*-genes in *Pseudomonas* had the highest integrity, including *alkBFGHJKL*, *alkN* and *alkST*. As a distinctive feature, almost all *alkF* and *alkN* were distributed in the species of *Pseudomonas*. The species in genus of *Marinobacter* located in clade B and the species in orders of *Rhodobacterales*, *Hyphomonadales* and *Maricaulales* located in clade A had relatively lower *alk*-genes integrity, these were *alkST* and *alkBGHJKL.* Most species in order of *Sphingomonadales* further lost *alkK*, and *alkT*. The *alkS* and *alkBGJH* were dominated in the genus of *Alcanivorax* and in order of *Hyphomicrobiales*. This phenomenon suggested that species barrier played an essential role in *alk*-genes dissemination, which needs to be broken in the transmission process.

**Figure 4:**
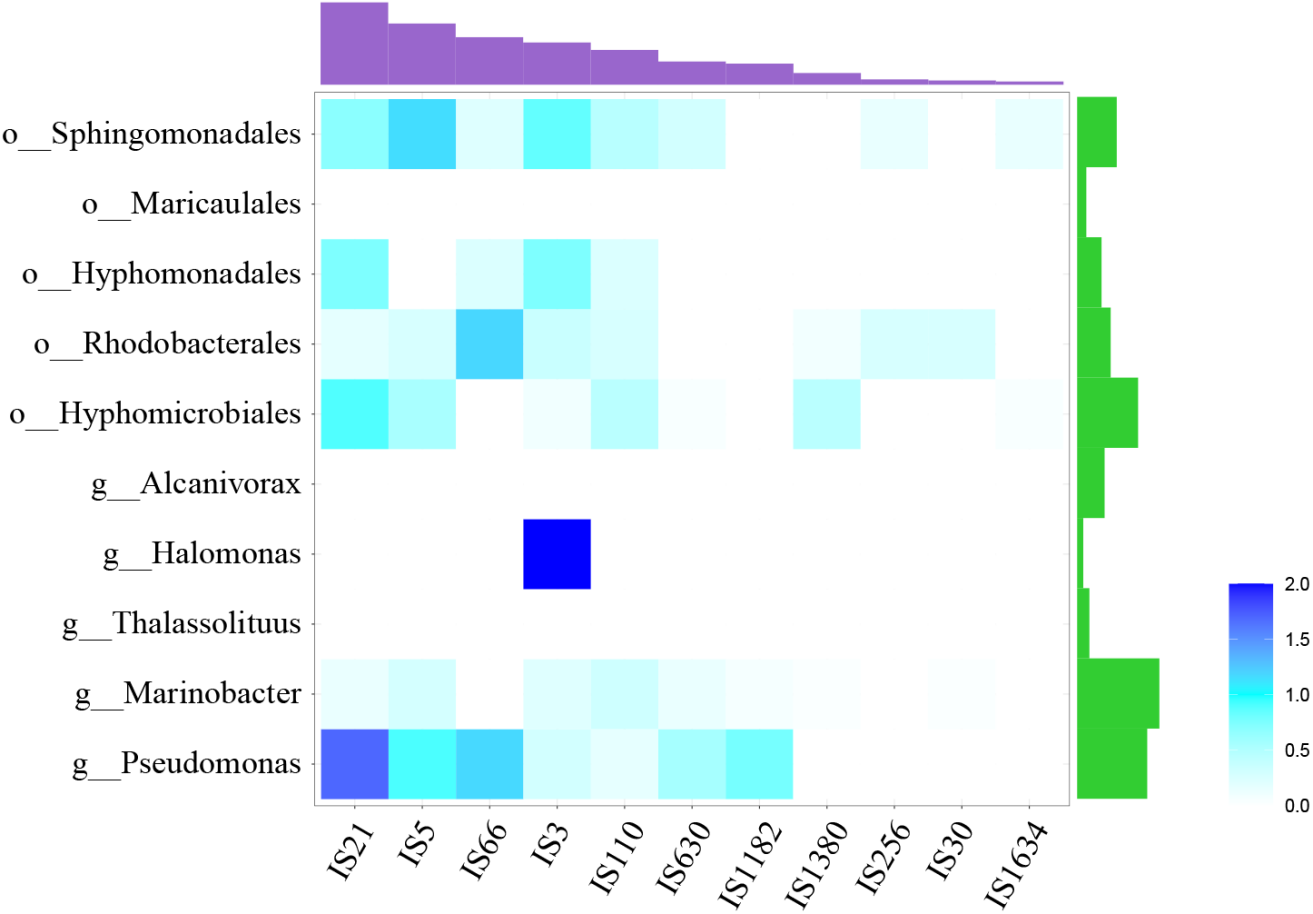
Distribution of IS elements. Heatmap showed the distribution of IS element, using IS element numbers normalized by *alk*-fragment number in each taxonomy. Amethyst bars at the top represented for the absolute number of each IS family. Green bars on the right represented for strain number of each taxonomy.

At the same time, many IS elements existed near *alk*-genes, as shown in Fig. 3. These IS elements belong to 11 families. Previous reports suggested that IS elements might play a role in mobilizing the *alk*-gene clusters (Van Beilen et al., 2001). We further explored the distribution of IS elements among different taxonomy. As shown in Fig. 4, IS elements are distributed unevenly in different taxonomy.

The *alk*-fragments from *Pseudomonas* and *Marinobacter*, *Hyphomicrobiales*, *Rhodobacterales*, *Hyphomonadales* and *Sphingomonadales* had amount of IS elements belonging to diverse families. The *alk*-fragments from *Halomonas* contained IS elements belonging to only one family, while almost no IS element was observed in *alk*-fragments from *Thalassolituus Alcanivorax* and *Maricaulales*. The results showed that in addition to IS elements, there might be other ways to transmit *alk*-gene clusters. In order to explore these factors for further research, larger scale data may be required.

## Discussion

In the present work, we performed a comprehensive investigation of *alk*-gene clusters focusing on their distribution, organization, phylogenetic relationship and dissemination potential. Using phylogenetic analysis methods, we characterized a novel alkane monooxygenase (*alkB*) clade, which was extracted from *alk*-gene clusters. According to our knowledge, this was the first comprehensive report on the survey of *alk*-gene clusters and their phylogenetic relationship in a mono-clade so far.

According to previous reports (Schneiker et al., 2006; Van Beilen et al., 2001), it can be inferred that *alk*-gene clusters were not limited to plasmids nor in *P. putida*. But their detailed distribution had not been reported. Results of this study showed that the *alk*-gene clusters can be observed only in *α*- and *γ*-proteobacteria. A mono-clade phylogenetic relationship of *alkB* extracted from *alk*-genes clusters was observed as shown in Fig. 2. This phenomenon was quite different from the ubiquitous distribution of *alkB* genes, from at least *α*-, *β*-, *γ*- and *δ*-proteobacteria, Actinobacteria, Bacteroidetes, and Spirochaetes (Nie et al., 2014; Wang & Shao, 2013).

The genes from *alk*-gene clusters performed co-evolution, which can be observed in Fig. 3 as a similar organization within closely related species. The closer the genetic relationship of species, the more similar the arrangement of genes in *alk*-gene clusters, indicating the restriction of interspecific transmission. Although the *alk*-gene clusters encode all proteins necessary for converting alkanes into corresponding fatty acids, the operon structures are not necessary for the function of alkane hydroxylase (Tsai et al., 2017).

The *alk*-gene clusters were observed on chromosome with higher frequency in this study, suggesting its transmission mechanism might not be dominated by plasmid transfer among species. These DNA fragments were relatively large (typically >20kb), resulting in difficulty in horizontal transfer between species. A positive correlation between the abundance of mobile genetic elements and the frequency of HGT was generally observed (Springael & Top, 2004). The horizontal transfer of large DNA fragments depends on large mobile elements. There were abundant IS elements in *alk*-gene clusters. These IS elements were relatively concentrated and focused on several special families, which might be responsible for the limiting factor of its spread among extensive species. But we observed almost no IS element in flanking regions of *alk*-gene clusters from *Thalassolituus Alcanivorax* and Maricaulales, suggesting other ways than IS elements for transmission of *alk*-gene clusters. Although no evidence is presented here, we suspect that the exchange of large fragments between genomes through homologous recombination is also a factor leading to the spread of *ALK* fragments.

The *alk*-gene clusters encode proteins involved in alkane degradation, which might make it possible for the host to obtain more energy sources. We observed extensive niches adaption of *alk*-gene clusters carrying strains. But the niches adaptions were more likely linked with the taxonomy of the host. Although the importance of host niches adaptability was not observed, we can infer that environment had less impact on the dissemination of *alk*-gene clusters. It was suspected that they had potential for dissemination in much wider ecological niches.

## Conclusion

In this study, a set of 120 *alk*-gene clusters was identified using a large-scale genome survey (>390,000 genomes). Phylogenetic, taxonomy distribution, niches distribution, organization structure, chromosome location and IS elements analyses were used to provide complete information about the *alk*-gene clusters. Although the *alk*-gene clusters were limited in *α*- and *γ*-proteobacteria, they had extensive niches distribution. At the same time, a large number of IS elements were observed nearby the *alk*-gene clusters. The benchmark dataset of *alk*-gene clusters established using genome-resolved approach in this study can provide base line for further investigation in metagenomic datasets, to explore its evolution and its position in ecology.

##  Supplemental Information

10.7717/peerj.14147/supp-1 Figure S1 Niches distribution of strains carrying *alk*-gene clusters along with the corresponding phylogenetic tree The phylogenetic tree was the same as in Fig. 1. The niches distribution is represented by the Check Mark on the right.

10.7717/peerj.14147/supp-2 Figure S2 *Alk*-fragments distribution on genome

10.7717/peerj.14147/supp-3 Figure S3 Taxonomic distribution of all available genomes included in this analysis The number of genomes from the top 15 phyla (A) and top 15 genera (B) was represented by bar size.

10.7717/peerj.14147/supp-4 Table S1 The GenBank assembly accession number of 117 genomes

10.7717/peerj.14147/supp-5 Supplemental Information 1 Newick tree files corresponding to Figure 1

10.7717/peerj.14147/supp-6 Supplemental Information 2 Newick tree files corresponding to Figure 2

10.7717/peerj.14147/supp-7 Supplemental Information 3 The accession numbers of prokaryotes genomes used in this study

## Additional Information and Declarations

### Data Availability

The following information was supplied regarding data availability: The prokaryotes genomes (both bacteria and archaea) accession numbers are available in the Supplementary File.
