# Supplementary figures and images for: A novel alkane monooxygenase (alkB) clade revealed by massive genomic survey and its dissemination association with IS elements
Source: PeerJ. 2022 Sep 28;10:e14147. doi: 10.7717/peerj.14147 (PMC9526415; doi:10.7717/peerj.14147)

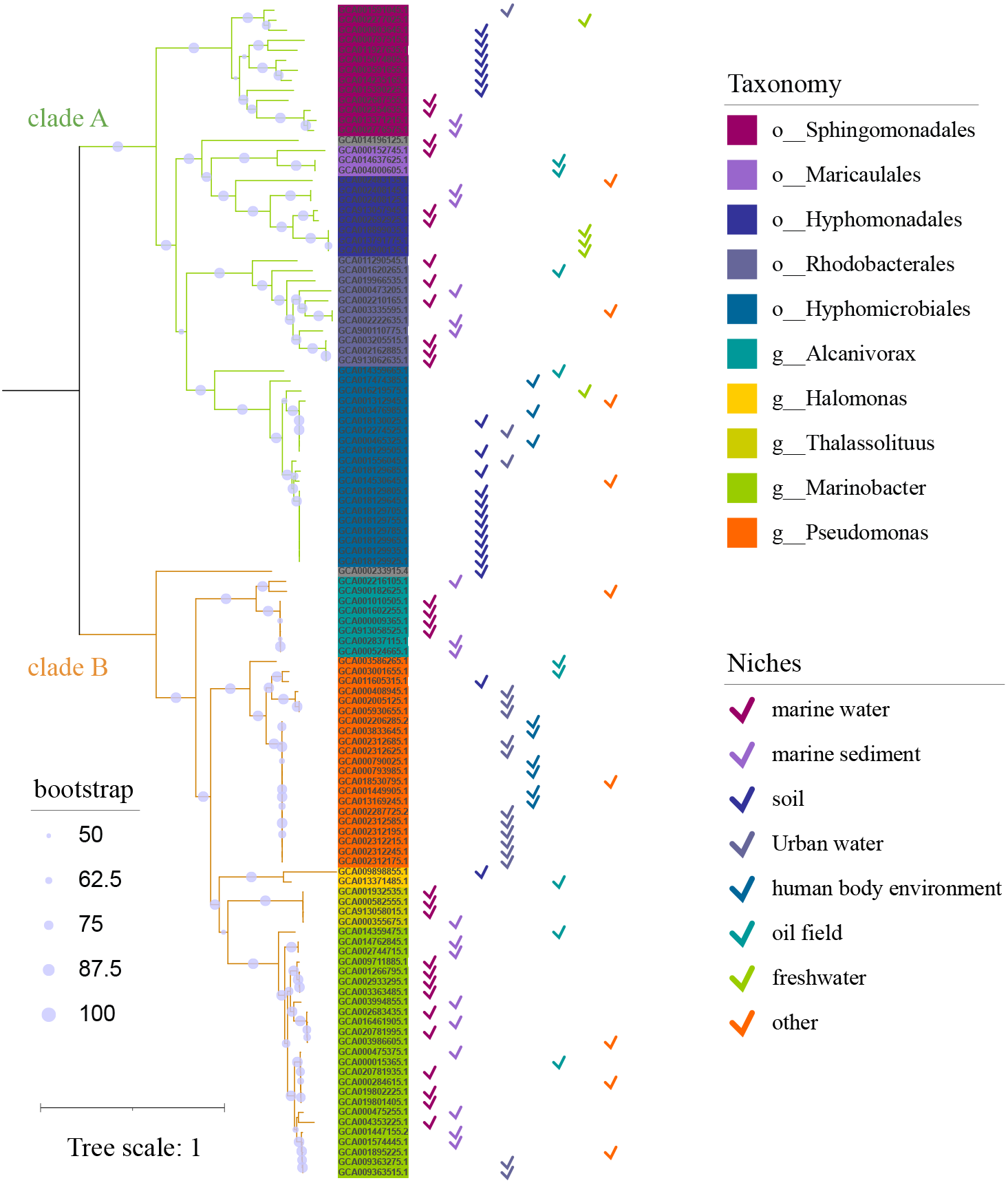

Supplement: Figure S1 — The phylogenetic tree was the same as in Fig. 1. The niches distribution is represented by the Check Mark on the right. [file peerj-10-14147-s001.png]

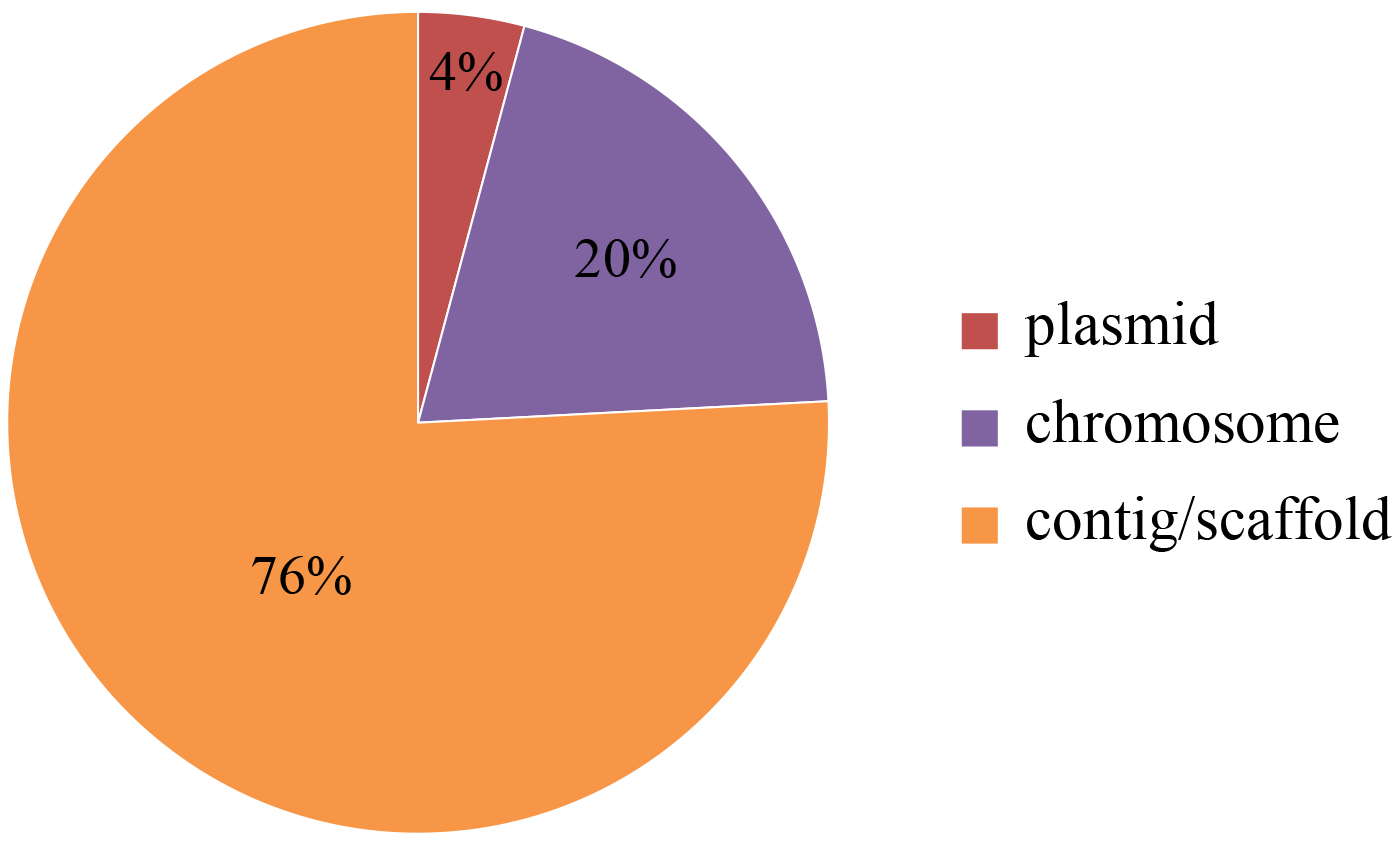

Supplement: Figure S2 [file peerj-10-14147-s002.png]

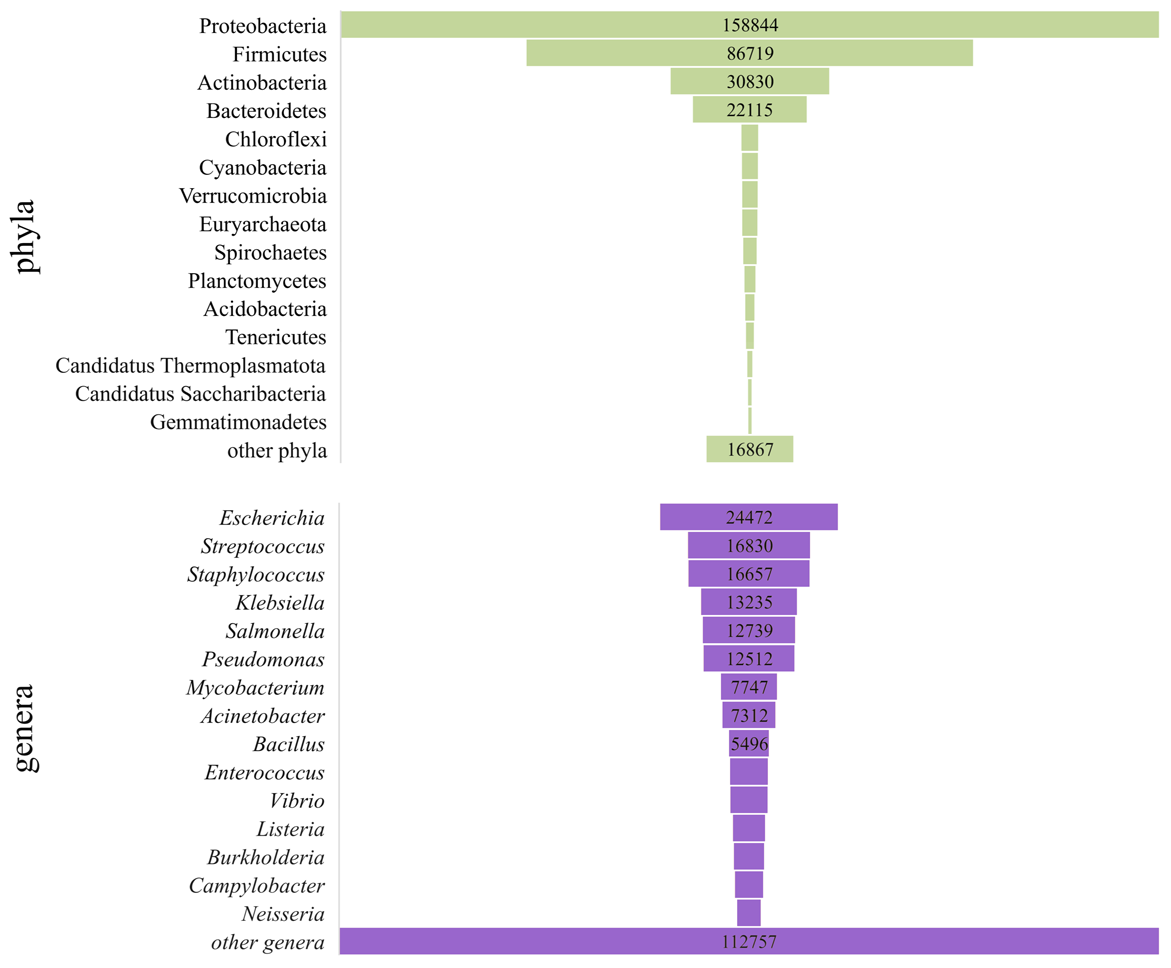

Supplement: Figure S3 — The number of genomes from the top 15 phyla (A) and top 15 genera (B) was represented by bar size. [file peerj-10-14147-s003.png]
